# Medical and Philosophical Intelligence

**Published:** 1814-08

**Authors:** 


### MEDICAL AND PHILOSOPHICAL INTELLIGENCE.

ROYAL SOCIETY.—On Thursday, the 26th of May, a paper, by Sir Everard Home, bart. on the Effect of different Injuries in the Brain upon Sensation, was read. The attempts to determine the functions of the different parts of the brain not having been attended with success, Sir Everard conceives that it would promote the advancement of physiology if medical men were to collect together, and arrange the effects produced by different diseases or injuries of the brain. The present paper contains the result of his own practice. It is divided into ten sections. 1. On the effect of water accumulated in the ventricles. Water accumulated in the ventricles, even even to the amount of 6½ ale pints, does not destroy the faculties, provided the bones of the cranium be not united, and the head enlarge according to the accumulation. A curious case was related of a young man whose head had increased enormously, and who retained his faculties entire, except some inconveniences from the size and weight of the head. He was in his nineteenth year, and the head was 33 inches in circumference. When the bones of the cranium, being united, prevent the head from enlarging, the accumulation of water in the ventricles destroys the faculties, and produces idiotism and death. 2. On the effects of concussion. It occasions nausea and vomiting, giddiness, and apoplectic fits, which return at intervals for some time. 3. On the enlargement of the blood-vessels of the brain. It occasions violent headaches, watchfulness, and disorders of the bowels. The beating of the arteries of the brain has been supposed essential to the exertion of the senses; but John Hunter retained his senses after the heart had apparently ceased to beat. 4. On the extravasation of blood. It produces similar effects as the accumulation of water; coma, nausea, apoplexy. 5. On the effects of the formation of pus. It occasions melancholy, lowness of spirits, and mania, with incessant talking. 6. On the effects of external pressure. The depression of the scull occasions loss of memory, the incapacity of using the proper conversation, &c. all which disappear when the cause is removed. 7. Internal pressure from tubercles produces similar effects. 8. Hydatids in the brain occasion bowel complaints, and a comatose state of the rectum and bladder. 9. Wounds in the brain occasion no symptom whatever, nor the destruction of any of the faculties. When a fungous excrescence of the brain takes place through a hole in the scull, the œsophagus becomes so sensible as to prevent the swallowing of solids, from the pain which they occasion. 10 Injuries of the spinal marrow in the neck, occasion paralysis of all the parts of the body below the injury.

---

An account of the diuretic powers of the *Pyrola umbellata* of North America, was read at the last meeting of the Medico-Chirurgical Society. This plant has been long since used by the Indians, in cases supposed to arise from defective secretion of urine, and promises to be a valuable acquisition to the materia medica. It has the property of increasing the appetite, and has been found serviceable in some cases of dropsy.

---

The following is said to be a very efficacious application for rheumatism, and is known by the name of Sanchez's Balsam:—Aromatic soap, an ounce; spirit of lavender, 4 ounces; camphor, 2 ounces; essential oils of peppermint, canelle, lavender, muscade, quaffle, sassafras, of each 15 drops; acetic æther, an ounce. The editor of the *Journale Generale de Medecine*, from whom we take the account, considers the efficacy of the remedy to reside principally in the acetic æther.

 Mr.

Mr. WANT is authorized by Sir JOSEPH BANKS and Major RENNELL, to publish their decided conviction that his Medicine, (an account of which was given in our last Number,) and the Eau Medicinale, are the same, as far as they are enabled to judge from the appearance, taste, and smell. Several gentlemen conversant with the latter, who have seen Mr. Want's preparation at Sir Joseph's house, are of the same opinion.

---

We understand a case of the successful treatment of Trismus, has lately occurred at the Westminster Hospital. A robust young man was brought in with his jaw firmly locked, in consequence, as was supposed, of the bite of a dog, between the finger and thumb. It was resolved to try the effects of copious blood-letting. He was bled from the arm till he fainted, which was evidently followed by a relaxation of the jaw. Next day he was cupped behind the ears, and upon the back, till the same effect took place. The jaw now opened almost half an inch, and he was enabled to take some nourishment, but the contraction returned towards evening. The third day he was again bled from the arm *ad deliquium.* The disease was now so far removed, that he was able to make a hearty dinner of boiled salmon, and drink some porter. Hearing some conversation about bleeding him again the next day, to which he was always very averse, having no sense of his danger, he left the hospital without leave. It is known, however, that he perfectly recovered, and is now quite well. He lost in all about sixty ounces of blood. The only medicine he took was a grain of tartarized antimony with two grains of opium each night at bed-time. Though this may not be considered as a very decided case of that species of trismus which has almost invariably proved fatal, it at least affords encouragement to give the same remedy a more full trial than it has hitherto experienced.

---

*A Marvellous Tale of Spontaneous Combustion in a Woman, from Leroux's Journale de Medecine.*

Dr. PROUTEAU, at one of the meetings of the Sociëté de Medecine Pratique, communicated a very remarkable fact of spontaneous combustion. A woman, 28 years of age, excessively fat, and addicted to drinking of spirits, (the doctor could not have hit upon a better subject for this miracle,) was found on fire in her chamber, where nothing else was burning. The neighbours who came in threw water on the body, already deprived of life; and related, that as they entered they heard a noise of something frying. She was lying about three feet from the chimney, the fire of which was concentrated in the hearth. The body left upon the floor a layer of black grease; and a book, in which she had probably been reading, was found untouched by the flame. The face and tongue were entirely consumed and reduced to a coal. Beneath the left breast, which was in part destroyed by the combustion, was an opening three inches in diameter, by which the doctor introduced his hand into the chest, and touched seve[illegible]l ribs, which he broke with as much facility as if they were calcined, &c. He concludes his narrati[illegible]e by giving his opinion, opinion, that the combustion began in the internal parts, and that the clothes were burned secondarily.

---

Pemphigus is a disease of somewhat rare occurrence, and we believe has not very frequently been observed to prevail epidemically. Mons. PETIET has seen it assume this character in the village of Batterans, containing about 294 inhabitants, without any apparent cause for such an anomaly. It came on with the usual symptoms of fever, in addition to which the patients were affected with general tumefaction, and an insupportable degree of itching. On the third day, vesicles, varying in size from that of a grain of hemp-seed to that of a walnut, appeared on every part of the surface of the body, but more particularly upon the abdomen, the arms, and the thighs. They contained a transparent and inodorous fluid, were easily broken, and left a violet brown spot upon the skin, which soon disappeared. In slight cases the fever sensibly abated after the appearance of the eruption, and the cure was effected in a week. In some cases, typhoid symptoms supervened, but the author does not state that the disease terminated fatally in any instance. He gave an emetic on the accession of the disease; and, with respect to the after treatment, he says nothing more than that he employed the Aqua Ammoniæ Acetitis, blisters, diffusible tonics, and camphor, in cases requiring them, but that the emetic and nitrous draughts were generally all that was necessary. Out of 294 inhabitants, 35, principally children, were attacked with the disease.

---

The Hepar sulphuris has been successfully applied by M. BERTRAND to tettery eruptions (*affections dartreuse*). As the French term *dartre* implies a variety of affections, we subjoin the description of two cases where it was useful.

1st. *Dartre rougeante.*—A woman of 57, had for seven years past suffered from a continual itching in the external parts of generation. On examination, little ulcerations (*disposées en plaques*) were discovered, from which a corrosive ichor was discharged. Hip Batter. were employed, in which two drams of the medicine were dissolved, and afterwards an ointment composed of one third of Hepar Sulph. and two thirds of simple cerate.

2d. *Dortre squameuse* (scaly tetter).—A woman, three years since, was subject to eruptions sometimes on the back of the hands and feet, and sometimes on the face. These eruptions being principally on the face, their hideous appearance induced the patient to apply for advice. They appeared on the cheeks under the form of thick scales, at first white, then of an ash colour, going successively through all their periods to a state of desiccation, when they fell off to reappear. Two ounces of Hepar Sulph. were dissolved in a bath for the whole body. The ointment was used as before, though somewhat weaker.

---

*Singular Disease.*—In the country of the Nogays, a tribe of Tartars dwelling between the Black Sea and the Caspian, on the south side of the river Kuma, there still exists a very singular disease, which which is mentioned by Herodotus, and several of the other ancient Greek writers. Herodotus informs us, that when the Scythians were inhabitants of Asia, they advanced towards Egypt, but were prevailed upon by Psammetichus, king of that country, to desist. On their return through Syria, they plundered the temple of Urania, in the city of Askalon. In consequence of this, the goddess sent a *feminine disease* among them.

Reineggs is the first modern writer who mentions the present existence of this disease among the Nogays, who are at present subjects of the Russian empire. Count Potocki, when travelling along the Kuma, in 1798, met with an old man who had this disease. He informed us that such persons are called Coss; and that the disease is not unknown in Turkey, where those subject to it have received the same appellation. The disease, as far as it has been described by Reineggs and Potocki, is distinguished by the following symptoms. It only attacks old persons. The skin grows wrinkled, the beard falls off, and the person assumes completely the appearance of a woman. He becomes incapable of propagating his species, and his sentiments and actions lose their masculine character. In this state he is obliged to shun the company of men and to associate with women, whom he perfectly resembles. The disease is now rare.

---

The Gazette de Santé, dated the 21st of June, contains two cases of Laryngitis, *Angine Laryngée œdemateuse, Gonflement ou œdeme de la Glotte,* which occurred in the Hotel Dieu, during a cold, rainy, and very variable season. A young man, convalescent from fever, early in the morning took cold; at noon he complained of general uneasiness, slight chills, and heat in the bottom of the throat; at four o'clock felt acute pain in the larynx; *inspiration was performed with great pain*, *but no difficulty was felt in expiration;* the voice was shrill and tremulous, deglutition difficult, countenance florid, pulse contracted and frequent. Five leeches were applied to the neck, and afterwards a cataplasm. He was not relieved by these means, had no sleep, and his anxiety was very great. On the following day his symptoms had increased; countenance was of a livid hue; *expiration* tolerably easy. The patient held his head backward, to facilitate *inspiration,* which was performed with *inexpressible anguish ; the pulse was hard, small, and frequent.* Five leeches and a blister were applied. He died at two o'clock. After death, the face, neck, and head, were suffused with blood. On dissection, the *mucous membrane of the epiglottis, glottis, and ventricles of the larynx, were inflamed and thickened.* The cellular texture anterior to and at the root of the epiglottis was so large, that the latter was pushed back toward the opening of the larynx, which was much contracted ; the trachea was healthy. If we had not such frequent specimens of the want of energy in the practice of the French physicians, we should suspect that man of idiotcy who could suffer inflammation of the larynx to run its course without the employment of blood-letting. If any disease require require depletion to its fullest extent, it is this in its acute state, which makes such rapid strides towards its fatal termination. The unutterable anguish, suffusion of the countenance, and gasping for breath, ought to be sufficient to induce at least a trial of the remedy, even if the inflammatory character of the disease were not so distinctly marked. We observe in the same narrative, a striking confirmation of the inconsistency of the attending physician. A case of Angina Trachealis (croup), in an adult, is adduced for the purpose of instituting a comparison between the two affections so nearly resembling each other in name. This case, compared with the former, is not remarkable for intensity of suffering, or great danger; yet, on the fourth day of the disease, *fifteen leeches, and, on the fifth, twenty more were applied to the throat, with advantage.* Now, if thirty-five leeches are necessary for the cure of a case of *Angina Trachealis,* we are at a loss to conceive upon what principles of calculation our continental friend discovers ten to be sufficient for the cure of that horrid assemblage of symptoms characteristic of *Angina Laryngea.* The reporter very properly recommends bronchotomy in the latter, as the means of averting suffocation. It will be obvious, that if the larynx be obstructed so as to make the ingress of air into the lungs difficult or impossible, this operation is our only resource: our past experience does not indeed warrant an expectation of *cure* from it; but, so long as the opening in the trachea is preserved, the symptoms are very considerably abated, as might, *à priori,* be expected. We submit to the consideration of our brethren in the profession, whether it should not be performed before the disease has arrived at its acme: the suffering of the patient would in a great measure be removed *in limine,* and perhaps a source of irritation destroyed, which may have a tendency to aggravate the disease.

---

M. Hagstroem, in a man who died of dysentery, found the lower part of the ileum and the large intestines indurated and gangrenous on their internal surfaces.

---

M. le Gallois, in the Dictionary of the Medical Sciences, has considered the heart, with respect to its anatomical and physiological characters. After having given an exact description of the position, form, and structure of this organ, with the phenomena of its movements, he has been induced to discuss a question which has frequently excited the attention of physiologists, viz. the unequal capacities of the ventricles. Different explanations had been given, which on the subject were but little satisfactory, when M. Sabatier maintained that this inequality arose after death, from the accumulation of blood which took place in the right cavities at the last moments of life. He supported his opinion by the fact, that individuals who die of hemorrhage, from a rupture of the vena cava, have the two ventricles of the same capacity, &c. M le Gallois, with the view of solving this important question, has instituted many experiments. After cutting away the two auricles, the aorta and pulmonary pulmonary arteries, at their origin, he filled the ventricles with mercury, and then separately weighed the quantity requisite to fill each ventricle. In every case the right was found to be larger than the left, and the difference was sometimes so great, that it appeared difficult to account for its existence in the healthy state. But, considering that, from a cause analogous to that which produces the rigidity of the muscles subject to the will, after death, the ventricles might be so influenced, and the left being the strongest in muscular structure would be consequently contracted the most. He endeavoured to overcome this rigidity, by kneading the ventricle with his fingers, and he in fact succeeded in enlarging the capacity. Some of the animals whose hearts were employed in these experiments were destroyed suddenly, others from hemorrhage, and in all the cavity of the right ventricle exceeded that of the left: the only exception was in the rabbit, where the capacity of the left surpassed that of the right, but it is doubtful whether this is a universal appearance. In the fœtus the same disposition exists, which may be owing to the particular mode of its circulation. M. le Gallois thinks that the reason of two ventricles of unequal capacities emptying themselves to the same degree during their sistole, is owing to a reflux of the blood of the right ventricle into its auricle, and which the arrangement of the value easily admits; the fact he conceives is incontestible.

M. le Gallois has also considered the circulation of the blood in the fœtus. According to M. Sabatier, the blood of the two venæ cavæ does not pass together through the foramen, but that of the inferior is directed by the Eustachian valve, and that of the superior enters directly the right ventricle, from whence it passes into the pulmonary artery by the arterial canal. M. le Gallois is of a different opinion: he conceives the Eustachian valve to be insufficient to perform this function; as in that case, instead of being placed at the anterior edge of the vena cava and the foramen, it ought to have been at the posterior; that it was sufficiently elevated to cover the greatest part of the diameter of the vena cava inferior, and that it was inclined towards the vein in the manner of an arch, through which the blood of the superior vena cava glides. The disposition of the Eustachian valve appears then most proper to favour the mixture of the blood coming from the two venæ cavæ, than to oppose such a union.

---

At a meeting of the *Société Philomatique* of Paris, on the 28th of May, 1814, Messrs. de Blainville and Magendie gave an account of Sir W. Adams's treatise upon Ectropium, &c. of which we made honourable mention in our Critical Analysis for April 1813. After noticing the leading features of the work, which they highly praised, Messrs. Blainville and Magendie concluded their report with very strongly expressing their admiration of the author's talents, and the benefit which he has conferred upon the profession; and recommended him as a corresponding member of the society. The proposition was unanimously adopted.

A large

A large biliary calculus was presented by M. Meglin to the *Athenée de Médecine* of Paris, which was extracted from the body of an old cavalry officer, in whom, during life, no symptoms were present that could lead to a suspicion of its existence. It completely filled the cavity of the gall bladder. The patient died of an organic affection of the *urinary bladder*, which had acquired an immense size, and ascended to the umbilicus. It was several lines in thickness, and its muscular membrane was entirely cartilaginous. During the latter years of his life, he suffered much from difficulty in passing urine. At length an abscess, preceded by inflammatory symptoms, was formed at the bottom of the bladder: when this broke, the effusion of urine and pus in the abdominal cavity produced visceral inflammation, which terminated in gangrene. He now complained of acute and burning pains in the abdomen, flatulency, inextinguishable thirst, and hiccup; his pulse was small, frequent, and contracted; respiration hurried; countenance livid, and extremities cold. In forty-eight hours death put a period to his sufferings.

Six other calculi, voided by a female long affected with hepatic colic, were also presented. The pain was sometimes so intense as to bring her into great danger, during a paroxysm of most acute insupportable pain, with icterus. Bleeding was had recourse to, after which she took Durand's dissolvent remedy (sulphuric æther and oil of turpentine) for six months. In this time she passed fifteen calculi, and was completely cured. M. Meglin speaks very confidently of the efficacy of the remedy. We believe M. Portal, in his treatise on Diseases of the Liver, speaks with some doubt on this point.

---

M. Zetherman has given an account of a dumbness occasioned by a calculous concretion, situated on the left of the frænum linguæ, and probably in the salivary duct. The patient was enabled to speak (at the age of fourteen years) after having spit out this stone, which was the size of a turkey-bean. The same gentleman found a tuft of hair in a vesicular tumour which he extirpated from the eyelid.

---

M. de Bierken had under his care a girl twelve years of age, whose tongue was so large that its point fell upon the chin, causing the eversion of the lower lip, and giving to the anterior teeth an horizontal direction. The deglutition had become difficult, the voice unintelligible, and the larynx projected. The disease had existed from the age of two years, but once was cured by replacing it (as we suppose, by confining it to its situation for a time). It was at length determined to extirpate it, which was done with complete success: the tongue was pierced by a needle with double ligature in two places; the space between the ligatures was first tied, and then the lateral portions; a part of the tongue was suffered to remain; she recovered her voice and deglutition; the lip retook its primitive situation; and the teeth, after being extracted, were replaced in a vertical direction, and properly secured.

 Dr.

Dr. SQUIRE will on Thursday, the 18th of August, begin a course of Lectures on the Theory and Practice of Midwifery, and the Diseases of Women and Children.

---

*In the Press, and will appear at the latter end of August,*

Facts and Observations on the Nature and Treatment of Liver Complaints, and Bilious Affections in general; deduced from long and extensive Practice in various Climates. The whole illustrated by Cases. By JOHN FAITHORN, formerly Surgeon in the Hon. East-India Company's Service.

